# Systematic Position of the Fossil Burrower Bug *Eocenocydnus lisi* Popov, 2019 (Hemiptera, Heteroptera, Cydnidae) Revealed by a Parallel/Cross-Eyed Viewing Method Used for Obtaining Three-Dimensional Images

**DOI:** 10.3390/insects14010022

**Published:** 2022-12-24

**Authors:** Jerzy A. Lis

**Affiliations:** Institute of Biology, University of Opole, Oleska 22, 45-052 Opole, Poland; cydnus@uni.opole.pl

**Keywords:** fossil bugs, Late Eocene, Isle of Wight, redescription, systematic position, new subfamily placement, new tribal placement, parallel, cross-eyed viewing method

## Abstract

**Simple Summary:**

The family Cydnidae (so-called burrower bugs) contains 76 fossil species primarily assigned to two subfamilies, Amnestinae and Cydninae. Only five fossil species were undoubtedly classified within the subfamily Sehirinae. *Eocenocydnus lisi*, described from the Late Eocene of the Isle of Wight, United Kingdom, was also tentatively placed in this subfamily. All other burrower bug subfamilies have no described fossil species to date. To verify the systematic position of *E. lisi* within the family Cydnidae, a parallel, cross-eyed viewing method was used. It is the first case incorporating this method into insect fossil morphological studies. It allows the recognition of many more morphological details than directly viewing a two-dimensional flat image.

**Abstract:**

The fossil burrower bug *Eocenocydnus lisi* described from the Late Eocene of the Isle of Wight, UK, is analysed using a parallel, cross-eyed viewing method. The species, tentatively placed in the subfamily Sehirinae, is redescribed and its systematic position is discussed. Newly recovered morphological characteristics allow it to be placed in the tribe Cydnini of the subfamily Cydninae.

## 1. Introduction

Cydnidae (so-called burrower bugs) contains 76 fossil species assigned to three subfamilies: Amnestinae, Cydninae, and Sehirinae [1,2,3,4,5]. Most of the fossil burrower bugs have been described in the first two subfamilies, and only five fossil species have been classified within the subfamily Sehirinae [1,2,3,4,5,6]. Additionally, *Eocenocydnus lisi* Popov, 2019, described from the Late Eocene of the Isle of Wight, UK, was tentatively placed also in this subfamily [7]. All other burrower bug subfamilies have not described fossil species yet [8].

In the present paper, this fossil genus *Eocenocydnus* Popov, 2019, is rediagnosed and its only species is redescribed. Moreover, a systematic position of this genus is discussed based on newly discovered characteristics not recognised during the original study [7].

## 2. Materials and Methods

This paper is based on the holotype of *E. lisi* deposited in the Museum of Isle of Wight Geology, Sandown, U.K. [7]. It was studied by me personally in 2009 when the late Prof. Y. Popov (Paleontological Institute, Russian Academy of Sciences, Moscow, Russia) asked for comments on this fossil burrower bug. However, my suggestions regarding its systematic position have not been considered in the original description. Unfortunately, I could not take a photograph of this fossil then. At present, this specimen is unavailable for personal examination [9]. Therefore, this study is based on the holotype photograph posted in the original paper [7], for which I have received permission for re-use from the Cambridge University Press. For its detailed redescription, a parallel, cross-eyed viewing method was used to obtain 3D images of the specimen [10,11,12,13,14,15]. Free-viewing methods are based on the fact that with practice, most people can view stereo pairs without using special devices [13,14].

**Figure 1:**
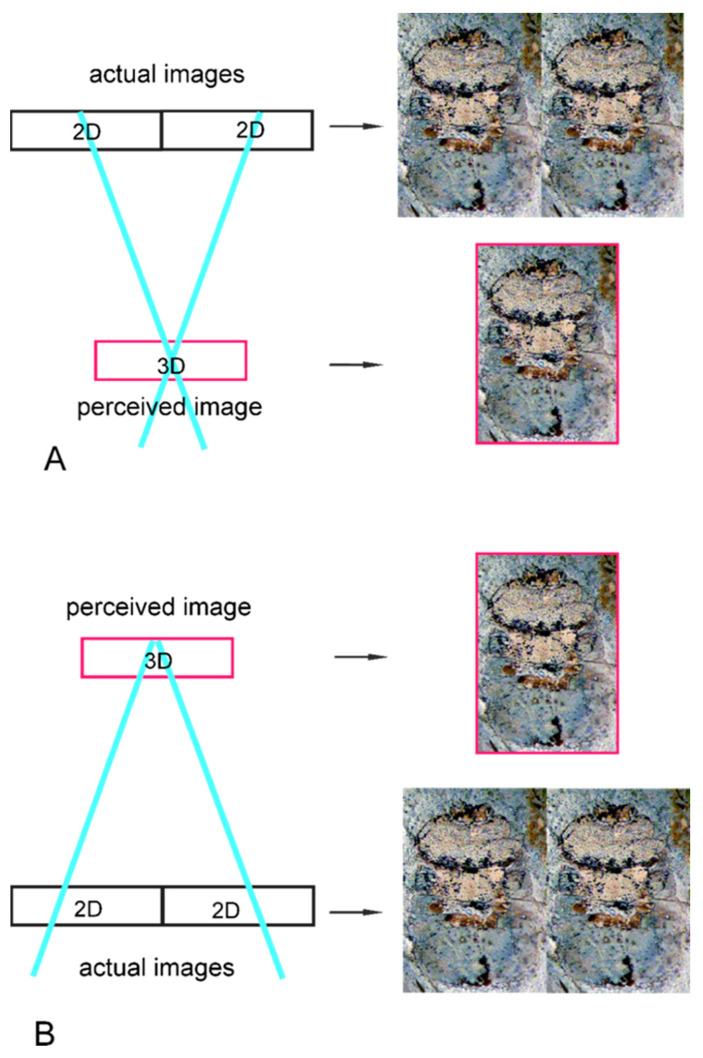
A comparison of the cross-eyed technique (**A**) versus the parallel viewing technique (**B**) for free viewing a stereo pair of images. Blue lines denote the left-eye and right-eye viewing lines.

There are two types of free viewing (Figure 1), distinguished by how the left and right eye images are arranged [13,14]. Some people can perform both types of viewing, some only one, while some, unfortunately, can do neither [13]; for a detailed description of how to look at flat images using both those types, see [13,14]. Most importantly, free-viewing methods allow the recognition of many more morphological details than when directly viewing two-dimensional flat images [13,14,15]. This is the first described case of using such a method for insect fossil morphological studies.

The scheme for the species redescription and a general morphological nomenclature follows Vršanský et al. [1], Lis et al. [2,4], and Imura [16]. The nomenclature for the cephalic chaetotaxy follows Lis and Pluot-Sigwalt [17], and for the metathoracic scent efferent system follows Kment and Vilímová [18]. The original photo of *E*. *lisi* habitus was appropriately corrected and improved using the Adobe Photoshop Elements editor version 10.0 (Adobe Inc., San Jose, CA, USA).

## 3. Results and Discussion

### 3.1. Redefinition of the Genus Eocenocydnus

**Figure 2:**
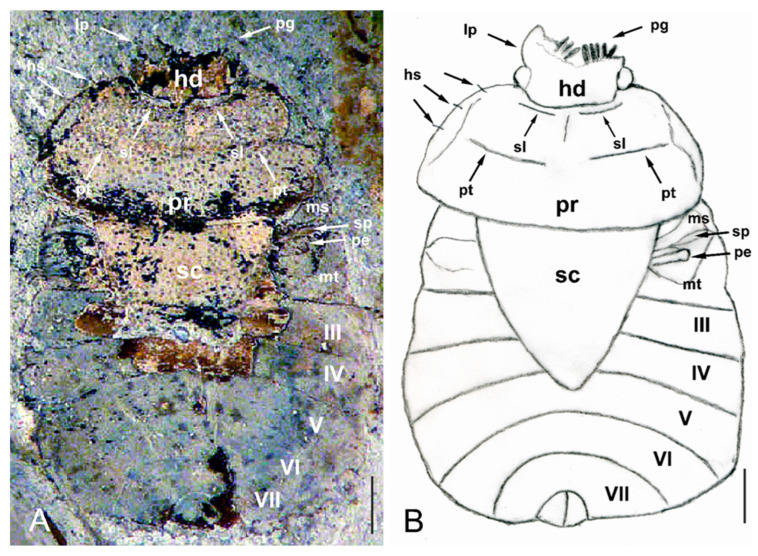
Holotype specimen of *E. lisi*, dorsal view. (**A**) Original photograph [7], modified; courtesy of the Cambridge University Press. (**B**) Line-drawing reconstruction (by J.A. Lis). Head (hd), hair-like setae (hs), lateral margin of left paraclypeus (lp), mesopleuron (ms), metapleuron (mt), peritreme (pe), pegs (pg), pronotum (pr), postmedian transverse depression (pt), scutellum (sc), submarginal impressed line (sl), metathoracic spiracle (sp), abdominal segments from III to VII (I–VII). Scale bars = 1.0 mm.

**Figure 3:**
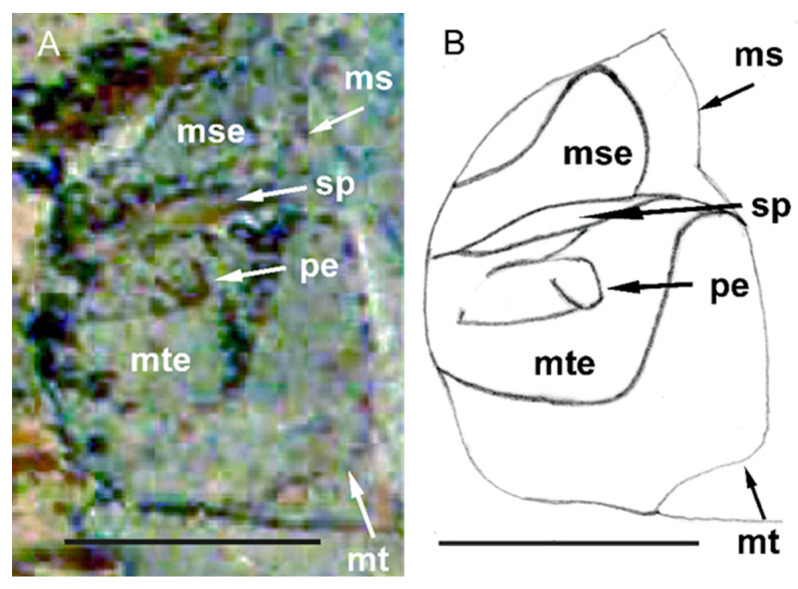
Holotype specimen of *E. lisi*, meso- and metathorax. (**A**) Original photograph [7], modified; courtesy of the Cambridge University Press. (**B**) Line-drawing reconstruction (by J.A. Lis). Mesopleuron (ms), mesopleural evaporatorium (mse), metapleuron (mt), metapleural evaporatorium (mte), *peritreme* (pe), metathoracic spiracle (sp). Scale bars = 1.0 mm.

Genus *Eocenocydnus* Popov (Figure 2 and Figure 3).

*Eocenocydnus* Popov, 2019: 55. Type species by original designation: *Eocenocydnus lisi* Popov.

Revised diagnostic characteristics. The body is large, at more 7.0 mm in length; its general shape is oval, being about 1.4 times as long as wide. The dorsal surfaces of the pronotum and scutellum are densely punctured. The head is spatulate; the paraclypei have a submarginal row of long pegs. He eyes are of moderate size, triangular, and slightly protruding. The pronotum is broader than it is long and is convex; its anterior margin is distinctly sinuated, the posterior is weakly concave, and its lateral margins are convex; its anterior angles are subacute, while the posterior ones are weakly rounded. The anterior submarginal impressed line on the pronotum is distinct; the pronotal disc is divided into lobes by a postmedian transverse depression. The lateral margins of the pronotum have submarginal setigerous punctures bearing hair-like setae. The scutellum is triangular and is broader than it is long. The evaporatorium on the mesopleuron is extensive, covering most of its surface; the metapleural evaporatorium is large, occupying about half of the metapleuron surface. The peritreme is rounded. The metathoracic spiracle is easily visible and elongated. The abdomen is broad.

### 3.2. Systematic Position of the Genus Eocenocydnus

As indicated in the original description of the genus [7], it was impossible to place this burrower bug in a definite subfamily due to its incomplete body preservation. Based on its large size and several other characteristics similar to those observed in the recent genus *Sehirus* Amyot et Serville, 1843, it was tentatively placed within the subfamily Sehirinae [7].

The newly discovered morphological characteristics using a parallel, cross-eyed viewing method seem slightly surprising. Two features, namely the presence of pegs on the head margins and hair-like setae on the lateral margins of the pronotum, are especially worth mentioning. Both identify the genus as not fitting into the subfamily Sehirinae.

**Figure 4:**
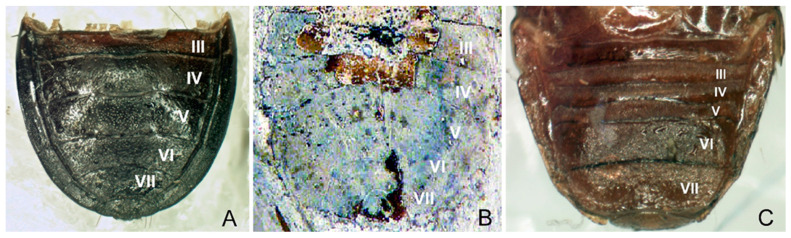
Abdominal segments III–VII, dorsal view. *Cydnus aterrimus* Forster, 1771 (Cydninae, Cydnini) (**A**), *Eocenocydnus lisi* (**B**), *Amnestus brunneus* Signoret, 1883 (Amnestinae) (**C**). (**A**,**C**) Original by J. A. Lis. (**B**) Courtesy of the Cambridge University Press, modified.

However, the lack of hemelytra does not allow for determining whether the claval commissure is present (the crucial characteristic for species classified within the subfamily Amnestinae) or absent (as in representatives of all other subfamilies). Nevertheless, a comparison of the morphological characteristics of the dorsal side of abdominal segments III–VII in species representing burrower bugs subfamilies suggests that *E*. *lisi* should not be placed within Amnestinae (Figure 4).

Additionally, the presence of the anterior submarginal impressed line on the pronotum; the triangular, broader than long scutellum; the shape of the evaporatoria on the meso- and metapleurons; and the shape of the peritreme indicate *Eocenocydnus* as belonging to the subfamily Cydninae (new subfamily placement). All these features also allow the genus to be classified within the tribe Cydnini (new tribal placement), close to *Cydnus* Fabricius, 1803; *Chilocoris* Mayr, 1864; and *Blaenocoris* J.A. Lis, 1997.

In light of the head shape, the presence of pegs on its lateral margins, the well-developed anterior submarginal impressed line on pronotum, and the shape of the evaporatoria on the meso- and metapleurons, *Eocenocydnus* is closely allied to *Chilocoris* [16,19,20,21,22]. As regards the shape of the peritreme, it is similar to the genus *Blaenocoris* [21]. The large body size [23,24] and the structure of the abdominal segments (Figure 4) suggests an affinity to species of the genus *Cydnus*.

### 3.3. Redescription of Eocenocydnus lisi

*Eocenocydnus lisi* Popov (Figure 2 and Figure 3).

*Eocenocydnus lisi* Popov, 2019: 57.

Redescription. The head is mutilated (however, a general outline of its left paraclypeus suggests its shape as similar to that of many *Chilocoris* species); its dorsal surface is impunctate; each paraclypeus has a submarginal row of about 5–6 long pegs; the vertex is more than five times wider than the eye. The pronotum is nearly twice as broad as it is long, and its dorsal surface is densely punctured; lateral parts of the pronotum are flattened in the anterior two-thirds, as in *Ch*. *deplanatus* J.A. Lis, 1997 [20]; their submargins have at least three setigerous punctures bearing hair-like setae. The mesopleural evaporatorium is large and well-defined; the metapleural evaporatorium occupies about half of the metapleuron’s surface. The peritreme is rounded, as in *Blaenocoris robustus* J.A. Lis, 1997 [21]. The metathoracic spiracle is elongated and easily visible. The abdomen is broad, and about 1.5 times as wide as it is long; the structure of the dorsal segments is similar to that described for species of *Chilocoris* and *Cydnus* (Figure 4).

Measurements (in mm). Body length 7.5; body width 5.5; head: length (impossible to estimate), width (across eyes) 1.6; eye width 0.25; vertex width 1.25; pronotum: length 2.25, widths 2.0 (anteriorly) and 4.5 (posteriorly); scutellum width 3.6; abdomen: length 3.5, width 5.5.

Material examined: Holotype: IWCMS.2012.574, female, part, Insect Limestone, NW Isle of Wight (IWCMS = Isle of Wight County Museum Service).

Remarks on the type material: The records for the IWCMS show that the specimen was borrowed by the late Yuri Popov (Paleontological Institute, Russian Academy of Sciences, Moscow, Russia) in 2009. The holotype number (MIWG DI.X 205.36) provided in the original paper was a loan number, not a specimen number. When most of the specimens were returned in 2012, the Isle of Wight County Museum did not have a curator; therefore, some specimens were not processed correctly. Therefore, it is unclear from the records whether they were accessioned into the collection, and if they were, they would have a new number. Things were complicated when some borrowers moved specimens between them and did not inform the museum. When it was returned, it was subsequently loaned out with a batch of specimens to be photographed. Therefore, the holotype specimen of *Eocenocydnus lisi* was unavailable for this study [9].

## 4. Systematic Palaeontology

Order: *Hemiptera* Linnaeus, 1758.

Suborder: *Heteroptera* Latreille, 1810.

Infraorder: *Pentatomomorpha* Leston, Pendergast et Southwood, 1954.

Superfamily: *Pentatomoidea* Leach, 1815.

Family: *Cydnidae* Billberg, 1820.

Morphological characteristics [8,17,18,23,24]. The body is strongly sclerotised, usually ovoid, and sometimes more or less elongated. Its length is from 2.00 mm to almost 20.00 mm. Its dorsal surface is usually more or less convex, although the dorsum is flattened in some genera. Most species are dark-coloured, being black or brown; only a few are brightly coloured with yellowish-brown or yellow markings. The dorsal body side is usually punctured with numerous more or less deep punctures, although sometimes it can be smooth, glossy, or shining without any puncturation. The head is usually broad, from semicircular to subtriangular; its lateral margins bear pegs, peg-like setae, or hair-like setae in various combinations. The eyes are small to large. Ocelli are usually present; however, sometimes they are almost invisible or absent. The antennae are predominantly five-segmented, but some species have only four antennal segments. The rostrum is four-segmented and varies in length. The pronotum is subquadrate, usually broader than long, and its lateral margins bear setigerous punctures armed with hair-like setae. The propleuron is characterised by a distinct punctured or smooth depression. The peritreme varies in shape and sometimes is strongly modified. The scutellum is more or less triangular or elongated.

The hemelytra vary in length; the corium is divided into the clavus and the meso- and exocorium. The claval commissure is absent or only occasionally present. The hemelytral membrane is usually typically developed or can be reduced and almost invisible. The costal margins are more or less convex and usually separated from the exocorium along the entire length; submarginal setigerous punctures bearing hair-like setae are present or absent. The metathoracic wings are well developed, although sporadically can be reduced with diminished venation. The abdominal trichobothria on sterna III to VII are oblique or longitudinal, varying in numbers (one or two) and patterns. The legs are solid and well-adapted for digging. The tarsi are three-segmented; although sometimes they are absent. The tibiae, especially the anterior tibiae, are more or less compressed and armed with numerous strong spines and setae. The tibial comb complex is well-developed and rarely absent. Coxal combs are always present, although in fossil specimens they sometimes are hardly visible.

Subfamily: *Cydninae* Billberg, 1820.

Morphological characteristics [8,17,18,23,24]. The body is dorsally more or less convex and dark-coloured. Only sporadically are the yellowish-brown or yellow markings present on the pronotum and corium. The dorsal side of the head, the pronotum, and the corium are usually more or less punctured; only occasionally are those body parts smooth and without any puncturation. The head is semicircular to subtriangular in outline and without any crenulations; the clypeal and paraclypeal submargins are armed with peg-like setae or hair-like setae, although a few also bear submarginal pegs. The eyes are small to large and more or less protruding; ocelli are usually present and well-developed to almost invisible or even absent. The antennae are five-segmented, although a few species have only four antennomeres. The rostrum varies in length and usually reaches the coxae of the middle pair of legs. The pronotum is broader than it is long and its lateral margins are armed with hair-like setae.

The peritreme varies in shape and is often strongly modified. The scutellum is triangular and not longer than it is broad or elongated. The claval commissure is always absent; the clavo-mesocorial suture is usually present, although sometimes it may be invisible or absent. Two abdominal trichobothria are present on sterna III–VII, usually posteriorly to the spiracle; the inner trichobothrium on sterna III–IV is sometimes placed anteriorly to the spiracle. Tarsi are always present; the second tarsal segment is subequal in diameter to the first and the third tarsomeres. The tibial comb complex is well-developed and consists of a row of a dozen to several dozen setae. Its two outer setae are distinctly or slightly longer than the remaining ones. Coxal combs are present on all legs, each composed of a regularly aligned row of more than a dozen gutter-like or scale-like setae. The metathoracic wings are usually well-developed; the first vannal vein (vannus I) is well-developed; the apical branch of the radial vein (R) is prolonged toward the outer wing margin and is at least partially separated from the median vein (M).

Tribe: *Cydnini* Billberg, 1820.

Diagnostic characteristics [8,17,18,19,20,21,22,23,24]. The morphological characteristics enabling the separation of the Cydnini species from the tribe Geotomini Wagner (the second tribe within the subfamily) are as follows:

- The body does not exceed 13.00 mm in length;
- The head margins are armed predominantly by pegs and peg-like setae, although hair-like setae are also present;
- The scutellum is short, triangular, usually not reaching the half-length of the hemelytra, and broader than or as broad as long;
- The peritreme is extended laterally in the form of a polished band, which is sometimes strongly recurved at its end.

Genus: *Eocenocydnus* Popov, 2019.

Species: *Eocenocydnus lisi* Popov, 2019.

Diagnostic characteristics ([7], present study). The body length exceeds 7.0 mm. The paraclypeal submargins of the head are armed with long pegs. The anterior submarginal impressed line on the pronotum is well-developed; the pronotal disc is divided into lobes by a postmedian transverse depression. The scutellum is triangular and broader than long. The evaporatorium on the mesopleuron is well-developed and covers most of the mesopleural surface; the metapleural evaporatorium is large and occupies about half of the metapleuron surface. The peritreme is rounded.

Remarks. *Eocenocydnus* is the only fossil genus of the tribe *Cydnini* known to date.

## 5. Conclusions

1. The fossil burrower bug genus *Eocenocydnus*, placed in the original description [7] tentatively within the subfamily Sehirinae, was transferred to the Cydninae tribe Cydnini.
2. A parallel, cross-eyed viewing method used for the first time in insect fossil morphological studies allowed the recognition of more morphological details than when directly viewing a two-dimensional flat image.

## Data Availability

Not applicable.

## References

[1] Vršanský P., Lis J.A., Schlögl J., Guldan M., Mlynský T., Barna P., Štys P. (2015). Partially disarticulated new Miocene burrower bug (Hemiptera: Heteroptera: Cydnidae) from Cerová (Slovakia) documents occasional preservation of terrestrial arthropods in deep-marine sediments. Eur. J. Entomol..

[2] Lis J.A., Lis B., Heiss E. (2018). *Chilamnestocoris mixtus* gen. et spec. nov., the first burrower bug (Hemiptera: Pentatomoidea: Cydnidae) in Upper Cretaceous Burmese amber. Cretac. Res..

[3] Wang Y.J., Du S.L., Yao Y.Z., Ren D. (2019). A new genus and species of burrower bugs (Heteroptera: Cydnidae) from the mid-Cretaceous Burmese amber. Zootaxa.

[4] Lis J.A., Roca-Cusachs M., Lis B., Jung S.H. (2020). *Pullneyocoris dentatus* gen. et sp. nov. (Hemiptera: Pentatomoidea: Cydnidae), the third representative of the subfamily Amnestinae from mid-Cretaceous amber of northern Myanmar. Cretac. Res..

[5] Du S., Gu L., Engel M.S., Ren D., Yao Y. (2022). Morphological phylogeny of new Cretaceous fossils elucidates the early history of soil dwelling among bugs. Front. Ecol. Evol..

[6] Statz G., Wagner E. (1950). Geocorisae (landwanzen) aus den Oberoligocanen Ablagerungen von Rott. Palaeontogr. Abteil. A.

[7] Szwedo J., Drohojowska J., Popov Y., Simon E., Wegierek P. (2019). Aphids, true hoppers, jumping plant-lice, scale insects, true bugs and whiteflies (Insecta: Hemiptera) from the Insect Limestone (latest Eocene) of the Isle of Wight, UK. Earth Environ. Sci. Trans. R. Soc. Edinb..

[8] Schuh R.T., Weirauch C.H. (2020). True bugs of the World (Hemiptera: Heteroptera). Classification and Natural History.

[9] Munt M. (2022). Personal communication.

[10] Adams L.P. (1974). Stereoscopic viewing of image pairs with the naked eyes. Photogramm. Rec..

[11] Green N.M. (1983). Stereo viewing simplified. Nature.

[12] Green N.M. (1994). Stereoimages—A practical approach. Structure.

[13] McAllister D.F. (2002). Stereo and 3-D Display Technology. Encyclopedia of Imaging Science and Technology.

[14] Çöltekin A. (2006). Foveation for 3D Visualization and Stereo Imaging. Ph.D. Thesis.

[15] Ono H., Lillakas L., Wade N.J. (2007). Seeing double and depth with Wheatstone’s stereograms. Perception.

[16] Imura J. (2011). New and obscure species of the genus *Chilocorus* Mayr in eastern Asia, with the proposal of a “*nitidus*-group” concept (Hemiptera: Heteroptera: Cydnidae). Zootaxa.

[17] Lis J.A., Pluot-Sigwalt D. (2002). Nymphal and adult cephalic chaetotaxy of the Cydnidae (Hemiptera: Heteroptera), and its adaptive, taxonomic and phylogenetic significance. Eur. J. Entomol..

[18] Kment P., Vilímová J. (2010). Thoracic scent efferent system of Pentatomoidea (Hemiptera: Heteroptera): A review of terminology. Zootaxa.

[19] Lis J.A. (1994). Studies on Cydnidae of the Australian Region V. The genus *Chilocoris* Mayr in Australia, Tasmania and Moluccas (Heteroptera). Bonn. Zool. Beitr..

[20] Lis J.A. (1997). Studies on Cydnidae of the Australian Region. X. Two new species of the genus *Chilocoris* Mayr from New Guinea (Hemiptera: Heteroptera). Int. J. Invertebr. Taxon. Genus.

[21] Lis J.A. (1997). Three new Australian genera of burrower bugs with four new species (Heteroptera: Cydnidae). Pol. Pismo ent..

[22] Lis J.A. (1999). The genus *Chilocoris* (Heteroptera: Cydnidae) in Australia. Acta Soc. Zool. Bohem..

[23] Froeschner R.C. (1960). Cydnidae of the Western Hemisphere. Proc. U. S. Natl. Mus..

[24] Josifov M.V. (1981). Fauna Bulgarica 12. Heteroptera, Pentatomoidea.

